# The complete chloroplast genome sequence of *Styrax chinensis* Hu et S.Y. Liang (*Styracaceae*)

**DOI:** 10.1080/23802359.2020.1821822

**Published:** 2020-09-17

**Authors:** Yukun Tian, Yaoqin Zhang, Lili Tong, Xiaogang Xu, Yabo Wang, Xiaoyu Jiang, Hongchao Wang

**Affiliations:** a Co-Innovation Center for Sustainable Forestry in Southern China, College of Biology and the Environment, Key Laboratory of State Forestry and Grassland Administration on Subtropical Forest Biodiversity Conservation, Nanjing Forestry University, Nanjing, PR China; b State Environmental Protection Scientific Observation and Research Station for Ecology and Environment of Wuyi Mountains, Nanping, PR China; c School of Horticulture & Landscape Architecture, Jinling Institute of Technology, Nanjing, PR China

**Keywords:** *Styrax chinensis*, phylogenomics, *Styracaceae*, complete chloroplast genome

## Abstract

*Styrax chinensis* Hu et S.Y. Liang, is one of the few evergreen species of *Styracaceae* with fragrant flowers. Here, we characterized the complete chloroplast (cp) genome of *S. chinensis* using next-generation sequencing. The circular complete cp genome of *S. chinensis* is 158,502 bp in length, containing a large single-copy (LSC) region of 87,817 bp, and a small single-copy (SSC) region of 18,001 bp. It comprises 132 genes, including 8 *rRNA* genes, 37 *tRNAs* genes, and 87 protein-coding genes. The GC content of *S. chinensis* cp genome is 36.93%. The phylogenetic analysis suggests that *S. chinensis* is a sister species to *Styrax suberifolius* in *Styracaceae*.

*Styrax chinensis* Hu et S.Y. Liang is a rare evergreen member of *Styrax*, trees, 5–20 m tall. Trunk to 34 cm d.b.h, mainly distributed in tropical and subtropical lowland areas (Huang and Grimes 2003). It possesses high values for medicinal, ornamental, and timber (Liang 1981). Due to no complete cp genome was characterized for *S*. *chinensis*. We characterized the complete cp genome sequence of *S*. *chinensis* (GeneBank accession number: MT648752) based on Illumina pair-end sequencing to provide a valuable complete cp genomic resource.

Total genomic DNA was isolated from fresh leaves of *S. chinensis* grown in Qingxiu mountain (N 22.4711, E 108.2240), Nanning, Guangxi, China. The voucher specimen was deposited at the herbarium of Nanjing Forestry University (accession number NF2020091). The whole genome sequencing was carried out on Illumina Hiseq platform by Nanjing Genepioneer Biotechnology Inc. (Nanjing, China). The original reading was filtered by CLC Genomics Workbench version 9, and the clean reading was assembled into chloroplast (cp) genome with SPAdes (Bankevich et al. 2012). Finally, CpGAVAS (Liu et al. 2012) was used to annotate the gene structure and OGDRAW (Lohse et al. 2013) was used to generate the physical map.

The circular genome of *S. chinensis* was 158,502 bp in size and contained two inverted repeat (IRa and IRb) regions of 26,342 bp, which were separated by a large single-copy (LSC) region of 87,817 bp, and a small single-copy (SSC) region of 18,001 bp. A total of 134 genes are encoded, including 87 protein-coding genes (81 PCG species), 37 *tRNAs* gene (30 tRNA species), and 8 *rRNA* genes (4 rRNA species). Most of the genes occurred in a single copy; however, seven protein-coding genes (*ndhB*, *rpl2*, *rpl23*, *rps12*, *rps7*, *ycf2*, and *ycf15*), seven *tRNA* genes (*trnA-UGC*, *trnI-CAU*, *trnI-GAU*, *trnL-CAA*, *trnN-GUU*, *trnR-ACG*, and *trnV-GAC*), and four *RNA* genes (*4.5S*, *5S*, *16S*, and *23S*) are totally duplicated. A total of nine protein-coding genes (*atpF*, *ndhA*, *ndhB*, *petB*, *petD*, *rpl16*, *rpoC1*, *rps16*, and *rpl2*) contained 1 intron while the other three genes (*clpP*, *ycf3*, *rps12*) had 2 introns each. The overall GC content of *S. chinensis* genome is 36.93%, and the corresponding values in LSC, SSC, and IR regions are 34.77, 30.34, and 42.78%, respectively.

In order to show the position of *S. chinensis* in *Styracaceae*, we collected 36 *Styracaceae* cp genomes and three taxa (Symplocaceae, Actinidiaceae, and Clethraceae) as outgroups with sequenced cp genomes. Sequences were aligned with MAFFT (Rozewicki et al. 2019). A phylogenetic tree was constructed based on the maximum likelihood (ML) with MEGA version 7 (Kumar et al. 2016). The results show that *S. chinensis* was clustered with other families of *Styracaceae* with 100% boot-strap values (Figure 1). In addition, *S. chinensis* was highly supported to be a sister species to *Styrax suberifolius* in *Styracaceae*.

**Figure 1.**
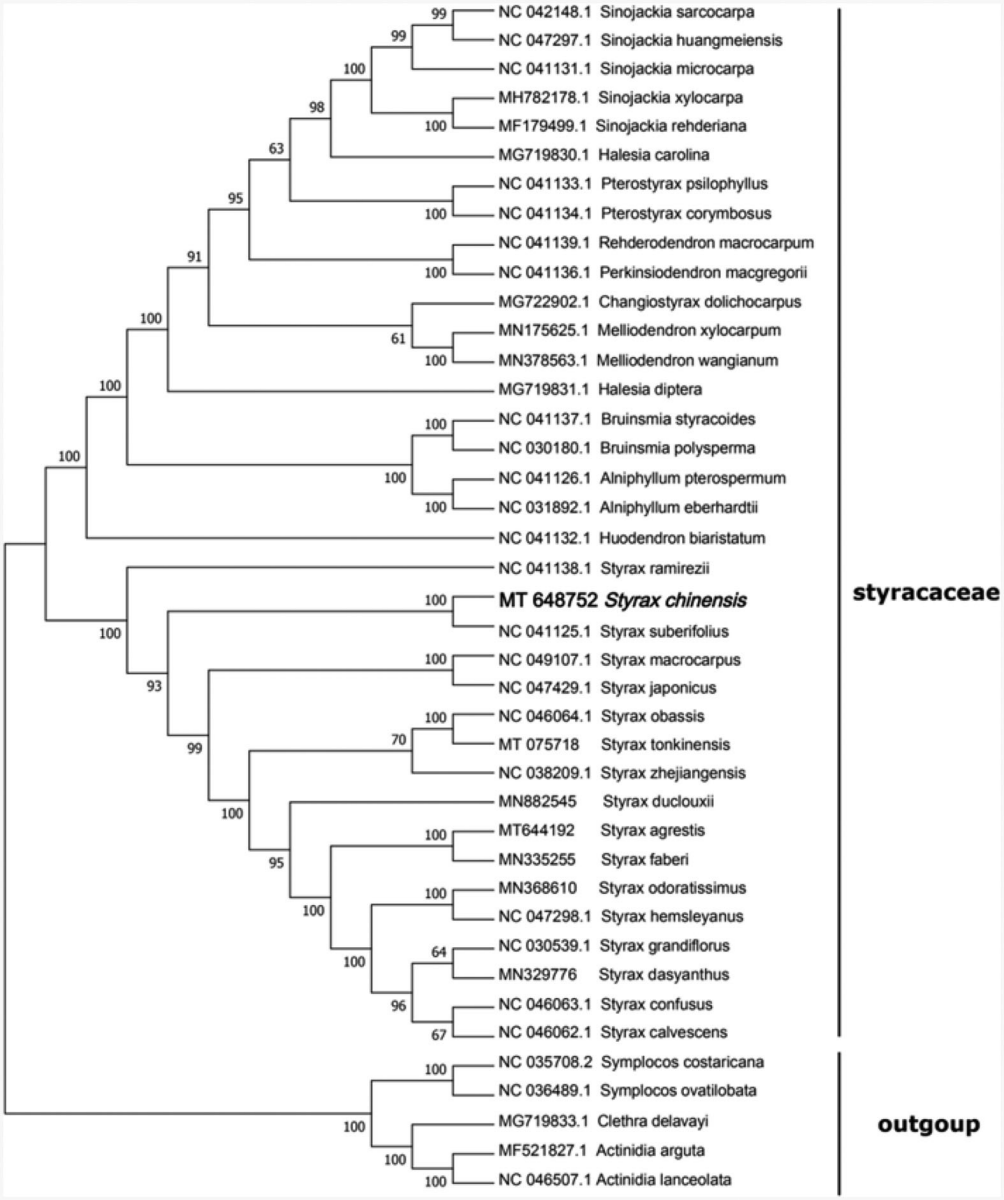
Maximum likelihood tree showing the relationship among *Styrax chinensis* and representative species within *Styracaceae*, based on whole chloroplast genome sequences, with 3 taxa from Ericales as outgroup. The bootstrap supports the values shown at the branches.

## Data Availability

The data is accessible from: https://pan.baidu.com/s/1GrqhmarC1h6WID-WPLwXdQ (password：rxqn).
